# NO PHONE, MORE STRESS: EXPLORING ANXIETY AS THE MEDIATOR BETWEEN NOMOPHOBIA AND STRESS

**DOI:** 10.1093/geroni/igae098.3677

**Published:** 2024-12-31

**Authors:** Rinanda Shaleha, Nelson Roque

**Affiliations:** Pennsylvania State University, University Park, Pennsylvania, United States; Pennsylvania State University, University Park, Pennsylvania, United States

## Abstract

This study (N=567) examines how nomophobia, the fear of being without one’s mobile phone, affects mental health by influencing anxiety and stress. Through regression and mediation analyses with nonparametric bootstrap, we found that anxiety mediates 69.24% of the impact of nomophobia on stress (ACME =.0724, 95% CI [.0568,.09], p <.00001). This substantial mediation underscores the central role of anxiety in the relationship between nomophobia and stress. Analysis by age and gender revealed variable mediation strengths: Young adults of both genders exhibited robust effects, with males showing a mediation proportion of 73.3% (ACME = 0.07424, p <.0001) and females 66.9% (ACME = 0.06940, p <.0001). Middle-aged females demonstrated a particularly high proportion at 88% (ACME = 0.05532, p =.002), contrasted by less pronounced effects in middle-aged males (ACME = 0.02218, p =.196). Older females also displayed significant effects, with a mediation proportion of 82.8% (ACME = 0.09425, p =.004). The observed demographic variations highlight the role of contextual factors such as age and gender in the psychological impacts of nomophobia. These insights are crucial for developing tailored interventions that more effectively counteract the adverse effects of mobile device dependency. Notably, the consistent mediation of stress by anxiety across most groups suggests that strategies focused on reducing anxiety could be particularly effective in managing nomophobia-related stress.

